# Finite-element simulation of photoinduced strain dynamics in silicon thin plates

**DOI:** 10.1063/4.0000059

**Published:** 2021-04-15

**Authors:** A. Nakamura, T. Shimojima, K. Ishizaka

**Affiliations:** 1 RIKEN Center for Emergent Matter Science, Wako, Saitama 351-0198, Japan; 2 Quantum-Phase Electronics Center and Department of Applied Physics, The University of Tokyo, Bunkyo, Tokyo 113-8656, Japan

## Abstract

In this paper, we investigate the femtosecond-optical-pulse-induced strain dynamics in relatively thin (100 nm) and thick (10 000 nm) silicon plates based on finite-element simulations. In the thin sample, almost spatially homogeneous excitation by the optical pulse predominantly generates a standing wave of the lowest-order acoustic resonance mode along the out-of-plane direction. At the same time, laterally propagating plate waves are emitted at the sample edge through the open edge deformation. Fourier transformation analysis reveals that the plate waves in the thin sample are mainly composed of two symmetric Lamb waves, reflecting the spatially uniform photoexcitation. In the thick sample, on the other hand, only the near surface region is photo-excited and thus a strain pulse that propagates along the out-of-plane direction is generated, accompanying the laterally propagating pulse-like strain dynamics through the edge deformation. These lateral strain pulses consist of multiple Lamb waves, including asymmetric and higher-order symmetric modes. Our simulations quantitatively demonstrate the out-of-plane and in-plane photoinduced strain dynamics in realistic silicon plates, ranging from the plate wave form to pulse trains, depending on material parameters such as sample thickness, optical penetration depth, and sound velocity.

## I. INTRODUCTION

Ultrafast light-induced strain dynamics in solid materials have attracted significant attention since the development of femtosecond laser sources. The generation and propagation of strains have been investigated in simple metals and semiconductors since the 1980s by employing ultrafast pump–probe measurements.^1,2^ Microscopically, strains in metals and semiconductors are mainly generated via two major mechanisms: thermoelasticity and deformation potential.^3^ When a material is irradiated by a pulsed light, the lattice temperature in the photo-excited region is increased by the electron–phonon coupling. As a result, thermoelastic strains are generated by instantaneous thermal expansion in many metals^4–8^ and semiconductors.^4,9^ Additionally, in semiconductors with band gaps, light also generates electron–hole pairs, thereby modifying interatomic forces. This means that the equilibrium positions of atoms are modified and strains are generated.^10,11^ Depending on the photo-excited electron distribution, the strain caused by the deformation potential can be either compressive or expansive, whereas thermoelasticity can only expand the lattice in usual materials that show positive thermal expansion coefficients.

In general, the dispersion of acoustic waves in finite size systems is modified compared to that in bulk samples by the traction-free boundary conditions at sample surfaces.^12^ For example, in plates made of isotropic media, longitudinal and transverse acoustic branches are transformed into two infinite sets of plate waves, which are referred to as symmetric and asymmetric Lamb waves.^12^ Because of the low-attenuation and high sensitivity to defects, these guided acoustic waves have been used for nondestructive inspection. In particular, the photoexcited acoustic waves have great advantage because they can be used without transducers that have to be attached to the sample.^13^ A pioneering experiment using time-resolved optical microscopy allowed researchers to visualize plate waves emanating from a point-source photoexcitation.^14^ Similar experiments on nano-fabricated materials (e.g., phononic crystals) have further revealed characteristic phenomena, such as acoustic rectification^15^ and the emergence of a phononic band gap.^16^ Furthermore, a zero-group-velocity Lamb wave, which can be used for many applications,^17^ was also observed at micrometer resolution.^18^ However, a point-source excitation generates pulsed acoustic waves that are composed of multiple modes and the interference between various modes with strong dispersion inevitably induce difficulty in the data interpretation in defect detection.^13^ In addition, sensitivity to the nanometer-size defect is severely limited by the wavelength of acoustic waves (i.e., the laser spot size, ∼1 *μ*m).^19^ In contrast, as described below, single- and few-mode acoustic waves can be photoexcited in the nanometric objects such as thin plates where the wavelength is determined mainly by the sample size (e.g., sample thickness in plates).

**FIG. 1.**
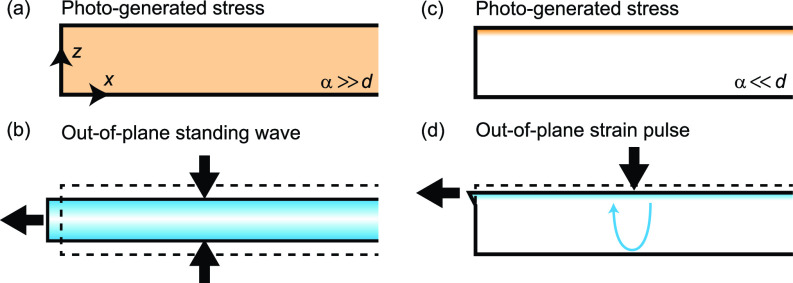
Schematics of photoinduced strain dynamics. (a) Approximately homogeneous stress distribution in the thin sample. The thickness *d* is much smaller than the optical penetration depth *α* in (a) and (b). (b) Out-of-plane standing wave. Contraction and expansion of the sample occur alternatingly as indicated by the thick black arrows. (c) Photo-generated stress localized near the surface region in the thick sample (α≪d). (d) Out-of-plane strain pulse generated at the surface that propagates along the thickness direction. The wave is reflected at the bottom surface of the sample as represented by the blue arrow.

Here, let us focus on nanometric thin plates. When thin plates are excited by optical pulses, complicated strain dynamics occur depending on their thickness. In the case where the optical penetration depth α is much greater than the sample thickness *d*, samples are approximately homogeneously excited, as shown in Fig. 1(a). The ultrafast carrier excitation and temperature increase induce stress and strain via deformation potential and thermoelasticity mechanisms. Depending on the sign of the stress, a sample may expand or contract in the out-of-plane direction [Fig. 1(b)]. Subsequently, the thickness of the sample periodically changes with the lowest-order acoustic resonance frequency *v*/2*d* in the form of a standing wave,^6,8^ where *v* is the sound velocity. Detailed Fourier transformation analysis of transient reflectivity measurements revealed that higher-order acoustic resonance modes with the frequencies of *mv*/2*d* (*m* = 3,5,7,…) are also excited in free-standing silicon membranes.^20^ In thick samples that satisfy *d* ≫α, on the other hand, carriers are only excited near the surface region [Fig. 1(c)]. It thus induces the strain dynamics significantly different from those in thin samples. Due to the localized stress, a strain pulse is generated at the surface and begins to propagate along the out-of-plane direction [Fig. 1(d)]. At the bottom surface of the sample, this strain pulse is reflected. Consequently, the pulse travels back and forth with the sound velocity *v*, referred to as “acoustic echoes,” whose time intervals are determined to be *T* = 2*d*/*v.*^4,9^ Theoretically, these out-of-plane strain dynamics can be modeled by temperature-carrier-strain equations, as described in the Methods section. The analytical/simulated solutions of these equations are known to agree well with experimental results presented in the literature.^4,6–11,20^

The photoinduced out-of-plane strains in plates are further converted into the in-plane strains when samples have any edges.^21^ Poisson's ratio of a material is typically positive; thus, the expansion (contraction) along the out-of-plane direction induces contraction (expansion) along the in-plane direction at the sample edges and defects [see Figs. 1(b) and 1(d)]. Simply speaking, the wavelengths of photogenerated acoustic waves in thin plates can be determined by the sample thickness and thus can be nanometer scale in very thin films. Nonetheless, the direct detection of acoustic waves in nanometer scale had been difficult due to the lack of experimental methods with nanometer and picosecond resolutions. Recent development of ultrafast electron microscopy enables us to investigate acoustic phenomena in this region. Indeed, in-plane acoustic waves with wavelengths of a few hundred nanometers have been observed in thin films by using ultrafast electron microscopy,^21–23^ in which only a few Lamb wave modes are excited. Therefore, the combination of short-pulse photoexcitation with nano-fabrication techniques could potentially facilitate the nanometer-scale contactless inspection. However, the detailed nature of photoinduced strains in plates, such as their wave shapes, amplitudes, and frequency characteristics, is still unknown due to the lack of theoretical and experimental investigations fully considering the effect of photoexcitation, as well as the velocity dispersion and the multiple-mode properties of plate waves. In particular, it is important to quantitatively analyze how the sample thickness and the optical penetration depth affect the transient characteristics of total strain dynamics.

In this study, we investigated carrier-strain dynamics in silicon plates with thicknesses of *d* = 100 and 10 000 nm through finite-element simulations. For the thin sample (*d* = 100 nm), it was determined that the sample was approximately homogeneously excited and that the out-of-plane standing waves were dominated by the lowest-order acoustic resonance mode because the optical penetration depth *α* (≃1000 nm) was much greater than *d*. The laterally propagating plate waves generated at the edge of the sample via the out-of-plane standing waves were mainly composed of two symmetric Lamb waves at the lowest-order acoustic resonance frequency. In contrast, for the thick sample (*d* = 10 000 nm), only the near-surface region was excited based on a mismatch between the optical penetration depth and sample thickness. As a result, the out-of-plane strain pulse propagating perpendicular to the sample surface was generated. The laterally propagating strains in the thick sample exhibited various branches and frequencies, including both asymmetric and symmetric Lamb waves.

## II. METHODS

The elastodynamic wave equation in isotropic and homogeneous materials, which is referred to as Navier–Cauchy equation, is written as

$$\rho \frac{\partial^{2}u}{\partial t^{2}}=\mu \nabla^{2}u+\left(\mu +\lambda \right)\nabla \left(\nabla \cdot u\right)+\nabla \sigma_{\mathrm{ext}},$$

where $u=(u_{x},\;u_{y},\;u_{z})$ are the atomic displacements, ρ is density, λ and μ are Lamè constants, and $\sigma_{\mathrm{ext}}$ is a photoinduced stress term. We define the *x* and *z* axes as the axes perpendicular and parallel to the thickness direction, and the origin is defined as the bottom-left edge of each sample [see Fig. 1(a)]. Therefore, *z* =* d* and *z* = 0 correspond to the top and bottom surfaces, respectively. We assume that the sample is infinite along the *y* direction. By assuming $u_{y}=0$ and $\partial u_{i}/\partial y=0$ (*i* =* x*, *y*, *z*), the two-dimensional elastodynamic wave equation is expressed as follows:^12^

$$\begin{array}{c} \rho \frac{\partial^{2}u_{x}}{\partial t^{2}}-\left(\lambda +2\mu \right)\frac{\partial^{2}u_{x}}{\partial x^{2}}=\lambda \frac{\partial^{2}u_{z}}{\partial x\partial z}+\mu \frac{\partial }{\partial z}\left(\frac{\partial u_{x}}{\partial z}+\frac{\partial u_{z}}{\partial x}\right)+\frac{\partial \sigma_{\mathrm{ext}}}{\partial x}, \end{array}$$ (1)

$$\begin{array}{c} \rho \frac{\partial^{2}u_{z}}{\partial t^{2}}-\left(\lambda +2\mu \right)\frac{\partial^{2}u_{z}}{\partial z^{2}}=\lambda \frac{\partial^{2}u_{x}}{\partial x\partial z}+\mu \frac{\partial }{\partial x}\left(\frac{\partial u_{x}}{\partial z}+\frac{\partial u_{z}}{\partial x}\right)+\frac{\partial \sigma_{\mathrm{ext}}}{\partial z}. \end{array}$$ (2)

In isotropic semiconductors, a stress $\sigma_{\mathrm{ext}}$ is mainly composed of two contributions from thermoelasticity and deformation potential. These contributions can be calculated from time-dependent lattice temperature $T_{l}(x,z,t)$ and electron–hole pair (carrier) density *n*(*x,z,t*). Light initially excites electron–hole pairs with an excess energy of $h\nu -E_{g}$, where hν is the excitation photon energy and $E_{g}$ is the band gap. We assume that the energy of such electron–hole pairs is released to the lattice through carrier–lattice coupling and Auger recombination processes.^24,25^ Based on previous studies,^4,6–11,20^ we define the following equations:

$$\begin{array}{c} C\frac{\partial }{\partial t}T_{l}-K_{T}\nabla^{2}T_{l}=\frac{E_{g}}{\tau_{R}}n+\frac{h\nu -E_{g}}{h\nu }P\left(z,t\right), \end{array}$$ (3)

$$\begin{array}{c} \frac{\partial n}{\partial t}-K_{n}\nabla^{2}n=-\frac{n}{\tau_{R}}+\frac{P\left(z,t\right)}{h\nu }, \end{array}$$ (4)

where *P*(*z*, *t*) is a source term. The definitions and values of other parameters are listed in Table I. The first terms on the right sides of Eqs. (3) and (4) denote Auger recombination processes, where energy is released to a third carrier and subsequently to the lattice. We assume that the electron–phonon and phonon–phonon relaxations are much faster than Auger recombination, and thus the lattice temperature $T_{l}$ is instantly increased after Auger recombination. It should be noted that the recombination time $\tau_{R}$ is dependent on the carrier density ($\tau_{R}^{-1}\propto n^{2}$).^24^ The stress term $\sigma_{\mathrm{ext}}$ is the sum of those by the thermoelasticity $\sigma_{\mathrm{TE}}$ and deformation potential $\sigma_{\mathrm{DP}}:$^3^

$$\begin{array}{c} \sigma_{\mathrm{TE}}=-3B\beta \left(T_{l}-T_{0}\right),\\ \sigma_{\mathrm{DP}}=-d_{\mathrm{eh}}n. \end{array}$$

The coefficients $B,\;\beta ,\;d_{\mathrm{eh}}$ are described in Table I. *T*_0_ = 297 K is the temperature before photoexcitation. It should be noted that positive and negative $\sigma_{\mathrm{ext}}$ values correspond to contraction and expansion of the lattice, respectively. We assume a simple profile for the source term P(z, t), which is defined as follows:^26^

$$P\left(z,t\right)=\frac{AF/\alpha }{\sqrt{2\pi }\Delta t}\text{exp}\left(-\frac{t^{2}}{2\Delta t^{2}}\right)\text{exp}\left(\frac{z-d}{\alpha }\right),$$

where *F*, Δ*t*, and *α* are the fluence, temporal width, and optical penetration depth of light, respectively. By using reflectivity *R*_ref_ and transmittance *T*_ref_, we define optical absorption rate *A* = 1 − *R*_ref_ − *T*_ref_. It should be noted that we use *AF* = 1 mJ/cm^2^ for all calculations despite the transmittance being dependent on thickness. This enables us to directly compare the calculation results for different thickness samples because the carrier and temperature distribution become almost identical. In addition, we ignore non-linear light absorptions such as two-photon and free-carrier absorptions in this model since these effects are weak and do not affect the calculation results under the present fluence of 1 mJ/cm^2^.^27^ By solving Eqs. (1)–(4) using the finite-element method, we investigated carrier-strain dynamics in silicon plates.

**TABLE I.** Parameters used for our simulations.

Symbol	Parameter	Value for Si	Unit	Ref.
ρ	Density	2.329	g/cm^3^	…
λ	Lamè constant	63.94	GPa	34, 35
μ	Lamè constant	50.85	GPa	34, 35
C	Heat capacity	$2.24\times 10^{6}$	J/(m^3^ K)	36
$K_{T}$	Thermal diffusion coefficient	$1.5\times 10^{5}\times T^{-1.226}$	W/(K m)	36
$K_{n}$	Carrier diffusion coefficient	$1.25\times 10^{-5}\times T$	m^2^/s	37
$E_{g}$	Band gap	1.12	eV	…
$\tau_{R}$	Auger recombination time	$n^{-2}/(3.8\times 10^{-43})$	S	24, 37
*B*	Bulk modulus	97.8	GPa	34, 35
β	Linear expansion coefficient	$2.6\times 10^{-6}$	K^−1^	38
$d_{\mathrm{eh}}$	Deformation potential parameter	$-2.3\times 10^{-24}\times B$	J	10
$T_{0}$	Temperature before photoexcitation	297	K	…
α	Optical penetration depth	971	nm	39
hν	Photon energy	2.4	eV	…
*AF*	Absorbed fluence	1	mJ/cm^2^	…
Δt	Optical pulse width	300	fs	…

A time-domain finite-element simulation was performed using the COMSOL Multiphysics software (version 5.4) with a partial differential equation module. To approximate semi-infinite silicon flat plates, we used two-dimensional models consisting of 16 *μ*m × 100 nm and 1600 *μ*m × 10 *μ*m plates of isotropic silicon for the thin and thick samples, respectively. The strains generated at one edge of each sample did not affect the results at another edge based on the adopted temporal scales. We used tetragonal mesh sizes of 2 × 2 nm and 200 × 200 nm for the thin and thick samples, respectively. The temporal discretization values were set to maximum values of 0.5 ps and 50 ps for the thin and thick samples, respectively. In the first 1 ps, where pulsed light with a duration of 300 fs was irradiated onto the sample, we used a maximum time step of 10 fs. The accuracy of the simulations, particularly during the first 100 ps for the thick sample, was carefully verified by testing different discretization values. All parameters used for our simulations are listed in Table I.

## III. RESULTS AND DISCUSSIONS

**FIG. 2.**
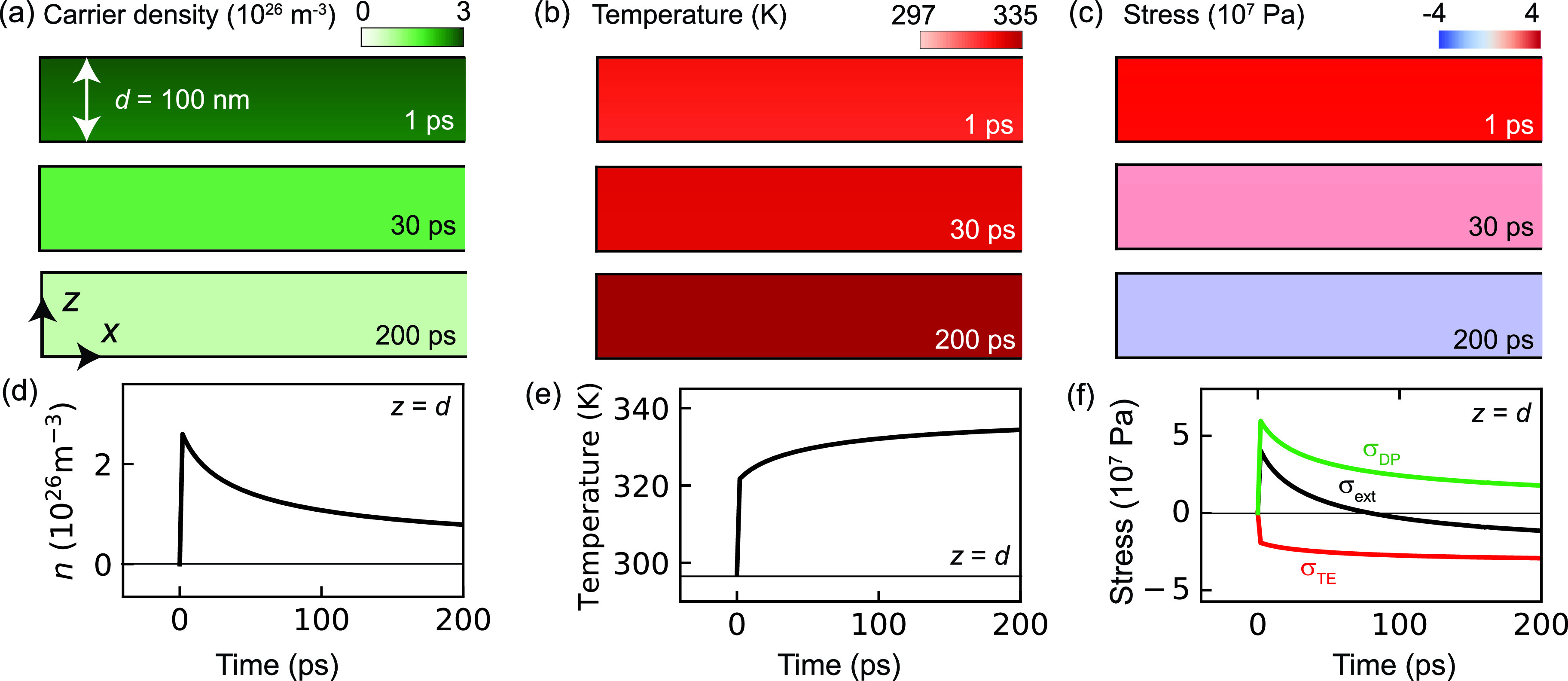
[(a)–(c)] Distributions of the carrier density n(x,z,t), temperature $T_{l}(x,z,t)$, and stress $\sigma_{\mathrm{ext}}(x,z,t)$ at 1, 30, and 200 ps for the thin (*d* = 100 nm) sample. The sample is photo-excited by a pulsed light propagating from the +*z* to −*z* direction with an absorbed fluence of 1 mJ/cm^2^ and 300 fs duration. Positive and negative $\sigma_{\mathrm{ext}}$ values corresponding to expansion and contraction, respectively. [(d) and (e)] Time dependence of *n*, $T_{l}$, and $\sigma_{\mathrm{ext}}$ at the top surface (*z* =* d*). $\sigma_{\mathrm{TE}}$ and $\sigma_{\mathrm{DP}}$ in (f) indicate the stresses caused by thermoelasticity and deformation potential, respectively.

Figure 2 presents the time dependence of the temperature $T_{l}$, photoexcited carrier density n, and stress $\sigma_{\mathrm{ext}}$ for a silicon plate with a thickness of *d* = 100 nm. As shown in Figs. 2(a) and 2(d), *n* rapidly increases to 2.5 × 10^26^ m^−3^ within the first 1 ps. The sample is approximately homogeneously excited since the sample thickness (100 nm) is much thinner than the optical penetration depth (≃1000 nm), as shown in Fig. 2(a). Following the first carrier excitation, the carrier density n gradually relaxes toward zero by the carrier recombination. The temperature $T_{l}$ increases to approximately 320 K within the first 1 ps [Figs. 2(b) and 2(e)] because we assume that the excess energy *hν* − *E*_g_ is instantaneously transferred to the lattice in Eqs. (3) and (4).^25^ On a longer timescale, the temperature increases gradually, which is associated with the carrier recombination. The resulting temporal profiles of the stresses $\sigma_{\mathrm{DP}}$ and $\sigma_{\mathrm{TE}}$ are presented in Figs. 2(c) and 2(f). It should be noted that positive and negative stresses cause contraction and expansion, respectively. In the case of silicon, $\sigma_{\mathrm{DP}}$ is positive, as indicated in previous studies.^10,28^ Therefore, the total stress $\sigma_{\mathrm{ext}}=\sigma_{\mathrm{DP}}+\sigma_{\mathrm{TE}}$ is positive within approximately 80 ps following photoexcitation and then changes to negative due to the thermal elasticity.

**FIG. 3.**
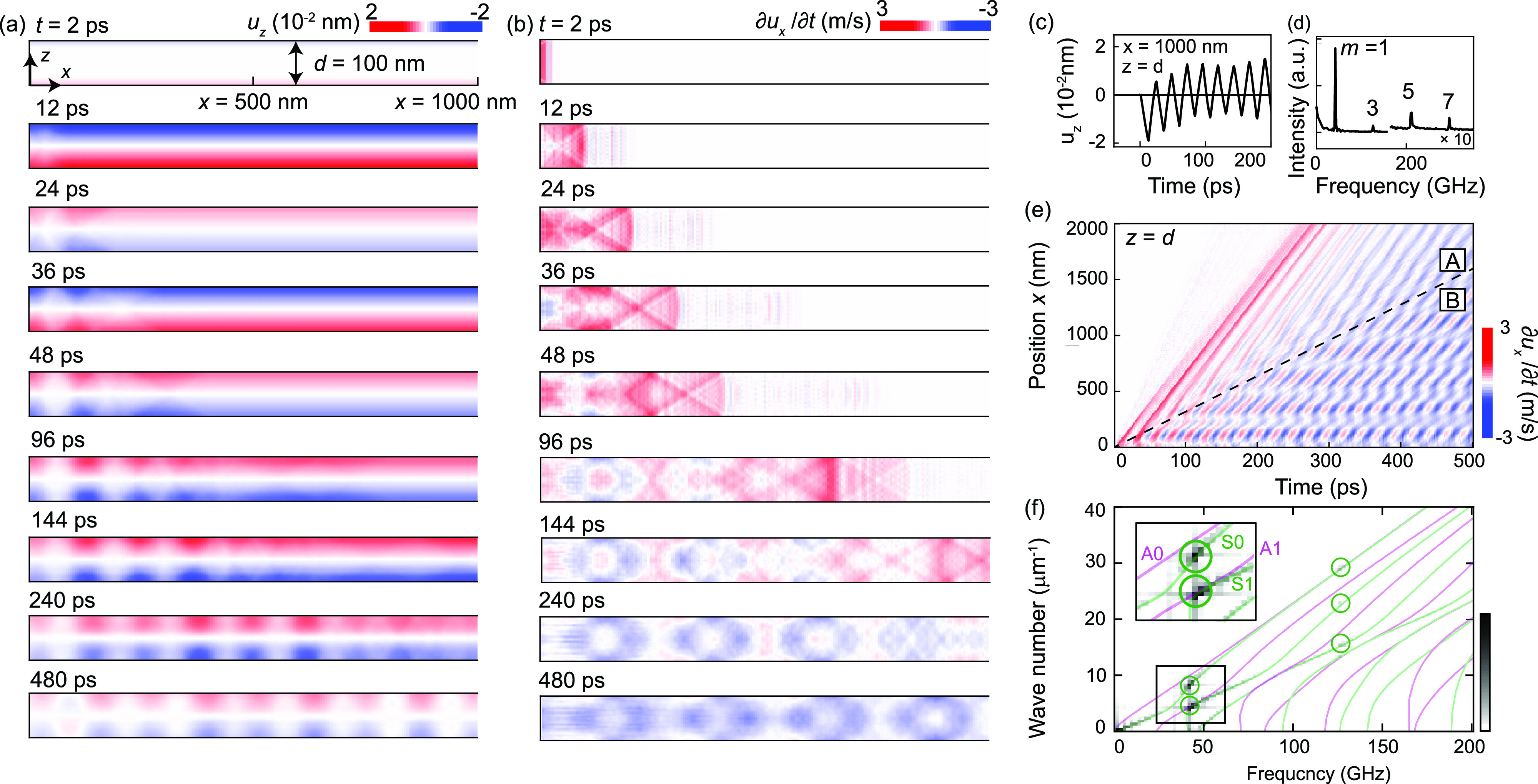
(a) Time dependence of *u_z_*(*x,z,t*) for the thin (*d* = 100 nm) sample. (b) Time dependence of $\partial u_{x}/\partial t$. The positive and negative values correspond to the contraction and expansion of the lattice along the horizontal direction, respectively. (c) *u_z_*(*x,z,t*) at *x* = 1000 nm and *z* =* d* = 100 nm. (d) Fourier-transformed version of the data in (c). The vertical axis is multiplied by 10 in the high-frequency region. (e) Space–time contour of $\partial u_{x}/\partial t$ at the top surface (*z* =* d*). The black dashed line is a guide that divides regions A and B. (f) Fourier-transformed version of the data in (e). The solid curves indicate the dispersion of the symmetric (green) and asymmetric (magenta) Lamb waves calculated using the frequency-Lamb equations. Strongly excited symmetric modes are highlighted by circles. The region indicated by the black rectangle is magnified in the inset.

In Fig. 3(a), we present the time dependence of the atomic displacement *u_z_* in the *x*–*z* plane. Because the stress term $\sigma_{\mathrm{ext}}$ is positive and homogeneous, the sample is contracted at 12 ps following photoexcitation, which is consistent with previous optical pump–probe experiments on silicon.^10,29^ Subsequently, alternating expansion and contraction, i.e., standing wave along the *z* axis, can be observed at *t* = 24, 36, and 48 ps. In a longer timescale, one can also see laterally propagating plate waves generated at the edge of the sample. We focus on the *u_z_* oscillation at *x* = 1000 nm and *z* =* d* (top surface) in Fig. 3(c), where the plate waves do not affect the *u_z_* dynamics until ∼300 ps. The obtained oscillation period of 24 ps corresponds to the lowest-order acoustic resonance mode under traction-free conditions, which satisfies *T* = 2*d*/*mv* (*m* = 1, 2, 3, …). The Fourier-transformed data of Fig. 3(c), which is shown in Fig. 3(d), reveal relatively weak contributions from higher-order (*m* = 3, 5, 7) resonance modes, as compared to the lowest (*m* = 1) mode. Since the sample is almost homogeneously excited, resonance modes with even *m* values, which exhibit asymmetric atomic displacement with respect to *z* =* d*/2, cannot be observed. Previous pump–probe experiments on free-standing silicon thin films (*d* = 221 and 346 nm) have also indicated the excitation of acoustic resonance modes with odd *m* values (1, 3, …, 19), where the amplitude was significantly suppressed as a function of frequency ($\propto 1/\omega^{2}$).^20^

Figure 3(b) presents the time dependence of *du_x_*/*dt* for the thin sample. We use the time derivative of *u_x_* to eliminate contributions of rather slow dynamics related to the thermal expansion in the lateral dimension. In the first 100 ps, the sign of *du_x_*/*dt* is mainly positive, indicating the presence of compression waves along the lateral direction driven by the positive stress $\partial \sigma_{\mathrm{ext}}/\partial x$. Similar to the case of out-of-plane standing waves, this positive stress is derived from deformation potential (carrier excitation), which also causes stress in the horizontal direction. Additionally, after *t* > 200 ps, periodic expansion and shrinkage of *u_x_* can be observed. These results are confirmed by the space–time contour of *du_x_*/*dt* for the top surface (z = *d*) in Fig. 3(e). Above the dashed line in Fig. 3(e) (region A), *du_x_*/*dt* is largely dominated by the periodic modulation with a period of 24 ps. Because this period is consistent with the lowest-order acoustic resonance frequency observed in Fig. 3(c), the periodic modulation of *u_x_* can be interpreted as plate waves coupled to the out-of-plane standing waves. In region B, *du_x_*/*dt* appears to be the superposition of several waves with different wavelengths but with oscillation periods equal to those in region A. These results indicate that the out-of-plane standing waves are converted into several branches of Lamb waves with different group velocities. By applying Fourier transformations along both the *x* and *t* axes, the distribution of photogenerated Lamb waves in the frequency–momentum space can be obtained, as shown by the gray color-scale in Fig. 3(f). Based on the frequency-Lamb equations,^12^ the analytical dispersion curves of the Lamb waves for isotropic silicon are also calculated and overlaid (green and magenta curves). Considering that the out-of-plane acoustic resonance modes are symmetric with respect to *z* =* d*/2 and cannot couple to asymmetric Lamb waves, it is natural that the intensity of the asymmetric Lamb waves (A0, A1) should be very weak. Therefore, the excitations at a frequency of approximately 40 GHz should be interpreted as the so-called lowest- and first-order symmetric Lamb waves (S0, S1) although it is difficult to distinguish S1 and A1 from Fig. 3(f). In addition to the strong excitation at 40 GHz, higher-order symmetric Lamb waves are also excited at 120 GHz, but their intensity is much weaker than those at 40 GHz, as also revealed in the frequency characteristics of the out-of-plane standing waves in Fig. 3(d). We note that the S0 mode excited in the low-frequency region (<25 GHz) is generated by the compressional waves driven by the stress term $\partial \sigma_{\mathrm{ext}}/\partial x$. These results indicate that the distributions of photogenerated Lamb waves are significantly affected by the amplitude of the out-of-plane acoustic resonance mode in Fig. 3(d) and their symmetry along the *z* direction.

**FIG. 4.**
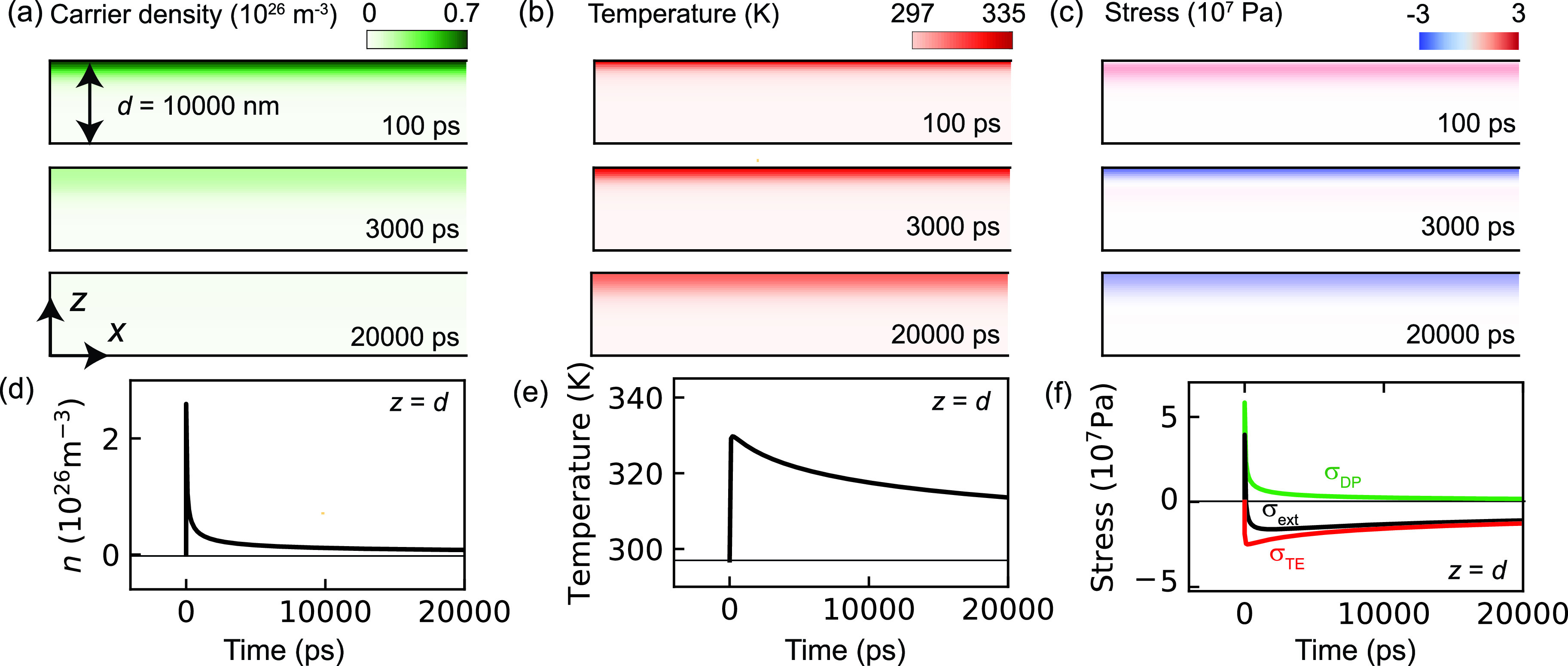
[(a)–(c)] Distributions of the carrier density n(x,z,t), temperature $T_{l}(x,z,t)$, and stress $\sigma_{\mathrm{ext}}(x,z,t)$ at 1, 30, and 200 ps for the thick (*d* = 10 000 nm) sample. The sample is photo-excited by a pulsed light propagating from the +*z* to −*z* direction with an absorbed fluence of 1 mJ/cm^2^ and 300 fs duration. Positive and negative $\sigma_{\mathrm{ext}}$ values correspond to expansion and contraction, respectively. [(d) and (e)] Time dependence of n, $T_{l}$, and $\sigma_{\mathrm{ext}}$ at the top surface (*z* =* d*). $\sigma_{\mathrm{TE}}$ and $\sigma_{\mathrm{DP}}$ in (f) indicate the stresses caused by thermoelasticity and deformation potential, respectively.

We now investigate the carrier-strain dynamics of the thick sample (*d* = 10 000 nm). We note that the spatial and temporal scales are significantly different from the case of *d* = 100 nm. Since the optical penetration depth of silicon is approximately 1000 nm, carriers are generated only near the top surface of the sample, as shown in Fig. 4(a). The excited carriers then relax to a quasi-equilibrium state via carrier recombination and carrier diffusion along *z*. The carrier recombination time is not significantly different from that of the thin (*d* = 100 nm) sample, and the time dependence of the carrier density at the top surface in Fig. 4(d) exhibits a sharp peak near *t* = 0. Similarly, the temperature near the top surface rapidly increases around *t* = 0 and then relaxes to a quasi-equilibrium state via thermal diffusion, although the temperatures at the top and bottom surfaces are still significantly different at *t* = 20 000 ps [Figs. 4(b) and 4(e)]. In Figs. 4(c) and 4(f), we present the profiles of the stress $\sigma_{\mathrm{ext}}$. At the top surface, a positive sharp peak in the stress can be observed around *t* = 0. The stress then rapidly drops to a negative value based on carrier recombination, which is similar to the case of the thin sample in Fig. 2(f). In contrast, the stress at the bottom surface is always small in this timescale. Such a strongly localized and asymmetric photoinduced stress with respect to *z* =* d*/2 induces the strongly pulse-like strain dynamics.

**FIG. 5.**
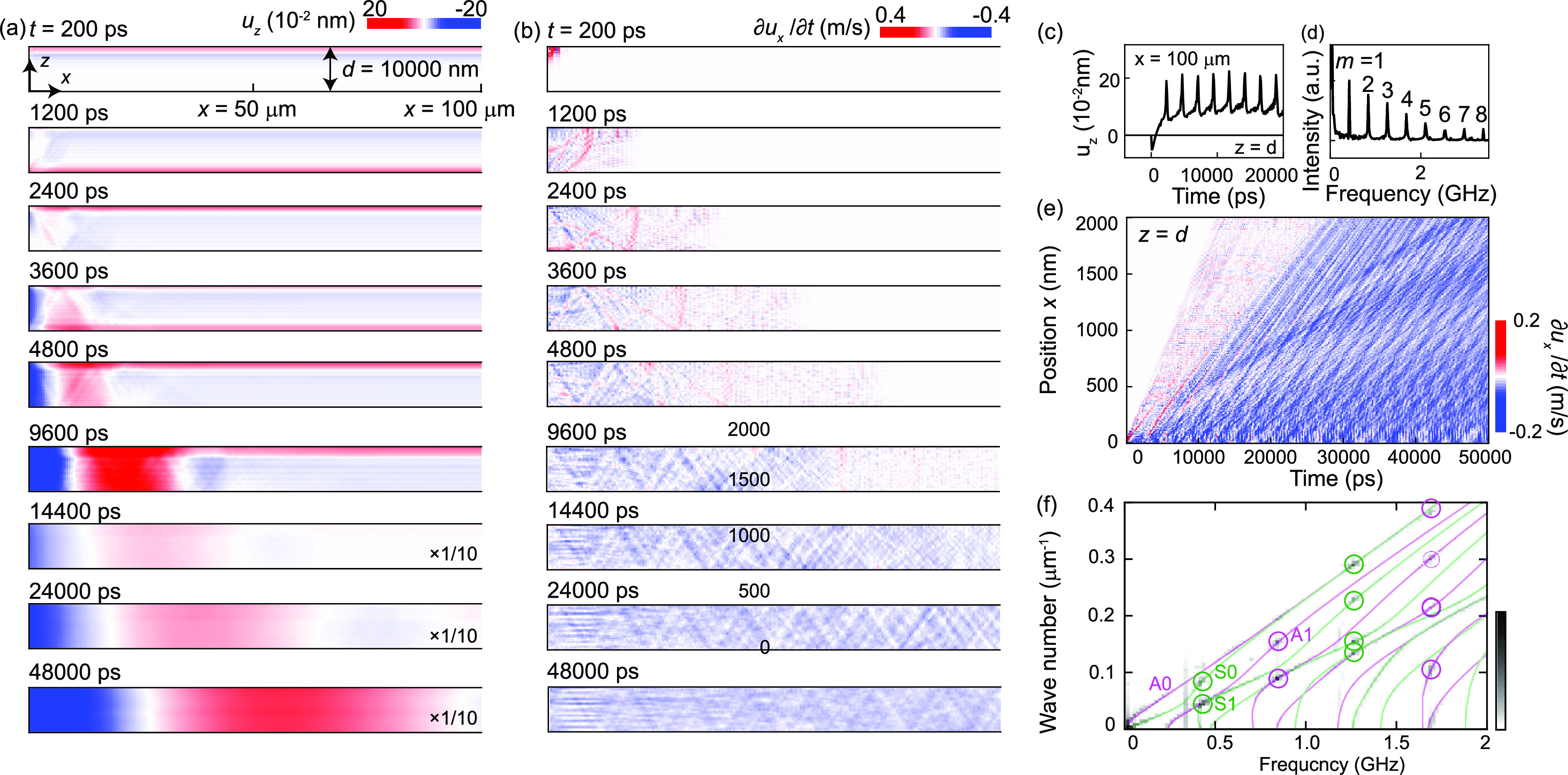
(a) Time dependence of *u_z_*(*x,z,t*) for the thick (*d* = 10 000 nm) sample. After 14 400 ps, the color scale is multiplied by 1/10. (b) Time dependence of $\partial u_{x}/\partial t$. The positive and negative values correspond to contraction and expansion of the lattice along the horizontal direction, respectively. (c) *u_z_*(*x,z,t*) at *x* = 100 *μ*m and *z* =* d* = 10 000 nm. (d) Fourier-transformed version of the data in (c). (e) Space–time contour of $\partial u_{x}/\partial t$ at the top surface (*z* =* d*). (f) Fourier-transformed version of the data in (e). The solid curves indicate the dispersion of the symmetric (green) and asymmetric (magenta) Lamb waves calculated using the frequency-Lamb equations. Strongly excited symmetric and asymmetric modes are highlighted by green and magenta circles, respectively.

Regarding the *z* direction, the localized stress term $\sigma_{\mathrm{ext}}$ causes acoustic echoes. As shown in Fig. 5(a), a strain pulse is generated near the top surface and then propagates toward the bottom surface with the bulk longitudinal sound velocity. At the bottom surface, the strain pulse is reflected and travels back toward the top surface (*t* = 1200 ps). Consequently, the *u_z_* value at the top surface shown in Fig. 5(c) exhibits periodic echo signals. The period of the acoustic echoes (2400 ps) is consistent with that of the lowest-order acoustic resonance mode (*T* = 2*d*/*v*), although it is determined by the round trip travel time in this case. We note that *u_z_* shows the strong *x*-dependent signals after 9600 ps in Fig. 5(b), in addition to the *x*-independent strain pulse. It suggests that the plate starts to bend because of the thermal expansion dominantly occurring at the top surface. In the frequency domain, the Fourier-transformed data in Fig. 5(d) exhibit acoustic resonance peaks for both even and odd *m* values due to the asymmetric stress $\sigma_{\mathrm{ext}}$. Additionally, the amplitude of the higher-order modes is not significantly suppressed compared to the thin (*d* = 100 nm) sample in Fig. 3(d).

Figure 5(b) presents the time dependence of *du_x_*/*dt* for the thick sample. Within the first 500 ps, a compression wave, which is characterized by positive *du_x_*/*dt* values, is generated at the upper-left edge of the sample via the instantaneous positive stress $\partial \sigma_{\mathrm{ext}}/\partial x$ and then propagates in the form of a cylindrical wave. This wave is gradually dephased because it is composed of a superposition of Lamb waves with different sound velocities. At 1200 ps, the compression wave is reflected at the bottom surface. The motion of the wave can be traced by following the positive (red) distributions. After 9600 ps, while the compression wave is almost completely suppressed by dephasing, *du_x_*/*dt* is dominated by the periodic changes induced by the out-of-plane strain pulse. It should be noted that thermal expansion generates a negative offset in *du_x_*/*dt* because the stress $\sigma_{\mathrm{ext}}$ is negative in this timescale [see Figs. 4(c) and 4(f)]. The periodic signal exhibits the series of triangular patterns in the *x*–*z* plane, which is synchronized with the reflection of the out-of-plane strain pulse at the top and bottom surfaces. The complicated space–time contour at the top surface (*z* =* d*) in Fig. 5(e) indicates that the laterally propagating strain pulses are composed of various Lamb wave modes. The Fourier-transformed data in Fig. 5(f) indicate that various modes are excited at the out-of-plane acoustic resonance frequencies, which are indicated by the circle markers. Unlike in the thin (*d* = 100 nm) sample, asymmetric Lamb waves are also generated at approximately 0.8 and 1.7 GHz. It should also be noted that the symmetric (asymmetric) Lamb waves are excited at the frequencies of odd (even) acoustic resonance modes, which are symmetric (asymmetric) with respect to *z* =* d*/2. Finally, the amplitude of the Lamb waves at the high frequencies is not significantly suppressed compared to the thin sample. These results agree with the frequency characteristics of the out-of-plane strain pulse. However, the signals from the in-plane strain pulses in Fig. 5(b) are much weaker than those from the thin sample [Fig. 3(b)], whereas the out-of-plane strain pulse has a larger amplitude. This is because the waveform of the strain pulse is significantly distorted by the velocity dispersion of Lamb waves.

The present finite-element simulations on thin and thick silicon plates demonstrate the transient dynamics of the out-of-plane and in-plane strains excited by pulsed light. Regarding the out-of-plane strains, the present results reveal the acoustic resonance mode in the thin plate, whereas the echoes of strain pulse traveling in the thick plate. These are more or less consistent with the previous model calculations.^4,6–11,20^ For the in-plane strains, we can now directly discuss the frequency- and wavenumber-profile as shown in Figs. 3(f) and 5(f), by explicitly considering the effect of optical penetration depth in our model. Such a discussion had been difficult in the previous finite-element simulation of in-plane strain on the WSe_2_ thin plate,^21^ which solely assumed the pulsed strain at the surface as the initial condition. In both the thin and thick plates, the distributions of the excited Lamb waves strongly reflect the symmetry and frequency characteristics of the out-of-plane strains, which is originally determined by photogenerated stress. The present calculation reveals that the plane wave like Lamb waves with the nanometer scale wavelength can be exited in a 100-nm sample. Such Lamb waves are difficult to be generated by the conventional point-source excitation and have great advantage in contactless and nondestructive inspection with nanometer spatial resolution. We expect that the present theoretical calculation in thin sample is verified experimentally by electron microscopy techniques in the future. Our model can be easily extended to more complicated systems, such as anisotropic, three-dimensional, and nano-fabricated systems, and thus will play an important role for future development of contactless nondestructive testing and development of ultrafast acoustic devices in nm scale.

## IV. CONCLUSION

We investigated the ultrafast carrier-strain dynamics of silicon thin (*d* = 100 nm) and thick (*d* = 10 000 nm) plates using the finite-element method. Approximately homogeneous photoexcitation in the thin plate sample generated an out-of-plane acoustic resonance mode at 40 GHz. As a result, the generated plate waves were largely dominated by two types of symmetric Lamb waves (S0 and S1) at 40 GHz and the contributions of asymmetric Lamb waves and higher frequency modes were heavily limited. In the thick (*d* = 10 000 nm) sample, only the near-surface region was excited based on the mismatch between the optical penetration depth and sample thickness. Localized stress generated echoes of the strain pulse in the out-of-plane direction, which were composed of multiple acoustic resonance modes over a wide frequency range. The resulting in-plane strain pulse in the thick sample was composed of various Lamb waves, including asymmetric and higher-order symmetric modes. These results indicate that photogenerated in-plane strains are significantly affected by the symmetry and frequency characteristics of out-of-plane strains, which was originally determined by the spatiotemporal profile of the photoinduced stress. Further investigations of additional microscopic strain generation mechanisms based on inverse piezoelectric effects,^30,31^ electrostriction,^32^ and the structural instability^23,33^ of anisotropic materials are promising research directions for controlling acoustic strains in nanometer and picosecond scales.

## Data Availability

The data that support the findings of this study are available within the article.
